# Non-invasively measured central and peripheral factors of oxygen uptake differ between patients with chronic heart failure and healthy controls

**DOI:** 10.1186/s12872-020-01661-4

**Published:** 2020-08-18

**Authors:** Joana Brochhagen, Michael Thomas Coll Barroso, Christian Baumgart, Jürgen Freiwald, Matthias Wilhelm Hoppe

**Affiliations:** 1 Institute of Movement and Training Science I, University of Leipzig, Jahnallee 59, 04109 Leipzig, Germany; 2 Department of Movement and Training Science, University of Wuppertal, Fuhlrottstraße 10, 42119 Wuppertal, Germany; 3 HELIOS Klinikum Wuppertal, University of Witten/Herdecke, Heusnerstraße 40, 42283 Wuppertal, Germany

**Keywords:** Activities of daily life, Alternative statistics, Arteriovenous oxygen difference, Clinical practice, Lactate, Questionnaire, Variability

## Abstract

**Background:**

Maximum oxygen uptake is an established measurement of diagnosing chronic heart failure and underlies various central and peripheral factors. However, central and peripheral factors are little investigated, because they are usually measured invasively. The aim of this study was to compare non-invasively measured central and peripheral factors of oxygen uptake between patients with chronic heart failure and healthy controls.

**Methods:**

Ten male patients with heart failure with reduced ejection fraction (62 ± 4 years; body mass index: 27.7 ± 1.8 kg/m^2^; ejection fraction: 30 ± 4%) and ten male healthy controls (59 ± 3 years; body mass index: 27.7 ± 1.3 kg/m^2^) were tested for blood pressure, heart rate, stroke volume, cardiac output, and cardiac power output (central factors) as well as muscle oxygen saturation of the vastus lateralis and biceps brachii muscle (peripheral factors) during an incremental cycling test. Stroke volume and muscle oxygen saturation were non-invasively measured by a bioreactance analysis and near-infrared spectroscopy, respectively. Additionally, a maximum isometric strength test of the knee extensors was conducted. Magnitude-based inferences were computed for statistical analyses.

**Results:**

Patients had a likely to most likely lower oxygen uptake, mean arterial pressure, and heart rate at maximum load as well as very likely lower isometric peak torque. Contrary, patients had a possibly to likely higher stroke volume and muscle oxygen saturation of the vastus lateralis muscle at maximum load. Differences in cardiac output, cardiac power output, and muscle oxygen saturation of the biceps brachii muscle at maximum load were unclear.

**Conclusions:**

Non-invasively measured central and peripheral factors of oxygen uptake differ between patients with chronic heart failure and healthy controls. Therefore, it is promising to measure both types of factors in patients with chronic heart failure to optimize the diagnosis and therapy.

## Background

Chronic heart failure is a clinical syndrome “caused by structural and/or functional cardiac abnormality, resulting in a reduced cardiac output” [1]. Based on the measurement of the ejection fraction, it can be distinguished between heart failure with reduced (< 40%), mid-range (40–49%), and preserved ejection fraction (≥50%) [1]. Heart failure with reduced ejection fraction occurs less often than heart failure with preserved ejection fraction and men are more affected than women [2]. In 2016, the disease was the second most common cause for hospital admission in Germany, whereby in more than 80% of all cases patients were older than 70 years [3]. The most recent European data present that 12-month all-cause mortality rates for patients with chronic heart failure were up to 17.4% [4]. Additionally, in 2012, costs for the disease in the USA were 30.7 trillion USD, which is predicted to increase by 127% by the year 2030 [5].

For the treatment of chronic heart failure, it is essential to accomplish valid diagnostic and therapeutic methods. Considering diagnostic methods, blood, electrocardiography, echocardiography, and maximum oxygen uptake are established measures [1, 6]. However, maximum oxygen uptake underlies various central and peripheral factors such as stroke volume, cardiac output, cardiac power output, and arteriovenous oxygen difference, respectively [7, 8]. In recent years, cardiac power output gained more importance, because of its possible superior prognostic impact compared to maximum oxygen uptake in patients with chronic heart failure [9–11]. Since it is a systemic disease, it also affects the peripheral system of the patients [12]. In fact, peripheral changes such as decreased skeletal muscle perfusion and mitochondrial dysfunctions in patients with chronic heart failure are partially seen as the main reason for a reduced performance during exercise [13, 14]. Additionally, the disease is characterized by a high heterogeneity especially affecting etiology and pathogenesis [15, 16]. As a consequence, factors underlying maximum oxygen uptake could differ between patients with chronic heart failure and thus cannot be generalized. Still, the aforementioned central and peripheral factors are often insufficiently addressed, because they are usually measured invasively [17]. Especially taken the importance of the cardiac power output and peripheral changes into account [9–11, 13, 14], it is promising to assess central and peripheral factors of oxygen uptake by recent non-invasive technologies such as bioreactance analysis and near-infrared spectroscopy [10, 17–19].

In this context, previous studies investigating patients with chronic heart failure have focused on stroke volume [20], cardiac output [10, 11, 20], and cardiac power output [10, 11]. Other studies examined healthy participants on stroke volume, cardiac output [21], and cardiac power output [22]. To get more insights into peripheral changes, two studies investigated isokinetic and isometric peak torque of the knee in patients with heart failure [23] and healthy participants [24], respectively. Overall, the studies show that patients with chronic heart failure have lower values of up to 45.8% in central factors and up to 35.3% in peripheral factors compared to separately investigated healthy participants. However, all previous studies only investigated either patients with chronic heart failure or healthy participants by different settings, questioning the validity of the described differences. Thus, for allowing stronger conclusions, more research comparing both groups by the same research design is needed.

To our knowledge, there is only one study that has compared the cardiac output between patients with chronic heart failure and healthy controls [25]. In this study, patients with chronic heart failure had a 32.9% lower cardiac output than healthy controls. Furthermore, regarding the known reduced oxidative capacity of the entire muscular system in patients with chronic heart failure [26], there is only one study that has investigated differences between the aforementioned groups [19]. The results showed no significant group differences in tissue oxygen saturation of the vastus lateralis muscle by a cycling ergometer. However, testing was carried out on a submaximal level. As maximum oxygen uptake is the gold standard for risk stratification of chronic heart failure [6], it is reasonable to investigate central and peripheral factors at maximum level as well. Taken together, while there are few studies that investigated central factors of maximum oxygen uptake in both groups by the same research design revealing significant differences, peripheral factors at maximum load are not investigated, yet.

The aim of this study was to compare non-invasively measured central and peripheral factors of oxygen uptake between patients with chronic heart failure and healthy controls. Based on previous research [19, 25], we hypothesize that patients with chronic heart failure show lower values in both central and peripheral factors than healthy controls. Our findings will increase the understanding of underlying factors of oxygen uptake in patients with chronic heart failure, which will help in diagnosis and therapy.

## Methods

### Participants

Ten male patients with chronic heart failure (62 ± 4 years) and ten male healthy controls (59 ± 3 years) participated. The patients were recruited from a local medical practice for cardiology, whereas the healthy controls were acquired of the investigators’ circle of acquaintances. Out of the ten patients, six were classified New York Heart Association (NYHA) class II, three NYHA class III, and one NYHA class IV. The mean ejection fraction and mean duration of the QRS complex of the patients were 30 ± 4% and 124 ± 28 ms, respectively. The relative medication was as follows: beta-blockers (100%); diuretics (80%); ACE inhibitors (80%); aldosterone antagonists (70%); AT1-antagnoists (20%); and Marcumar (30%). Besides their heart failure, the patients suffered from different comorbidities, namely arterial hypertension (40%), hypercholesterolemia (40%), bronchial asthma (20%), diabetes mellitus (10%), fatty liver disease (10%), chronic obstructive pulmonary disease (10%), renal failure (10%), allergies (10%), stroke (10%), and depression (10%). To describe the impact of chronic heart failure on daily situations, the Kansas City Cardiomyopathy Questionnaire was used. The maximum score is 100, whereas a higher score indicates a better condition [27]. The corresponding categories and scores reached were: physical limitation (77 ± 29); symptoms (87 ± 20); symptom stability (62 ± 29); social limitation (82 ± 21); self-efficacy (88 ± 15); and quality of life (78 ± 26). Further characteristics of both groups are presented in Table 1. Inclusion criteria for both groups were an age of 50–70 years and less than 3 h of exercise per week, so that both groups matched based on their activity level. Additionally, due to the definition considering heart failure with reduced ejection fraction, patients were included when an ejection fraction of ≤35% was present, as investigated before [18, 28]. This ensured that typical heterogeneity of chronic heart failure such as etiologies, demographics and co-morbidities was reduced [1]. Exclusion criteria for both groups were acute diseases speaking against maximum load testing and acute or chronic limitations of the motor system. All participants signed a written informed consent. The study was approved by the Ethics Committee of the local university (MS/BB 180321).

**Table 1:** Anthropometric characteristics, variables measured under maximum load, and isometric peak torque of the dominant leg

Variables	Chronic heart failure mean ± 90% CI	Healthy controls mean ± 90% CI	x-fold-SWD mean ± 90% CI	Indicator
Age [years]	62 ± 4	59 ± 3	1.7 ± 3.0	u
Height [cm]	178.4 ± 4.4	180.6 ± 4.6	−1.2 ± 2.9	u
Mass [kg]	87.8 ± 6.1	90.7 ± 5.9	−1.2 ± 3.6	u
BMI [kg/m^2^]	27.7 ± 1.8	27.7 ± 1.3	−0.1 ± 3.6	u
Fat [%]	27.6 ± 1.7	25.1 ± 1.7	3.5 ± 3.5	**
Fat-free mass [kg]	63.6 ± 3.7	67.8 ± 4.0	−2.7 ± 3.5	**
Skinfold thickness – M. vastus lateralis [mm]	9 ± 2	6 ± 1	4.8 ± 3.2	**
Skinfold thickness – M. biceps brachii [mm]	7 ± 1	6 ± 2	0.8 ± 1.9	u
P_max_ [W]	96 ± 17	201 ± 23	− 12.8 ± 3.1	****
VO_2rest_ [l/min]	0.5 ± 0.1	0.6 ± 0.1	−2.3 ± 3.2	u
VO_2max_ [l/min]	1.3 ± 0.2	2.5 ± 0.2	− 13.6 ± 3.1	****
VO_2max_ [ml/kg/min]	15.6 ± 3.0	28.0 ± 2.1	− 11.7 ± 3.7	****
HR_max_ [1/min]	133 ± 8	156 ± 12	−5.5 ± 4.0	***
RER_max_ [VCO_2_/VO_2_]	1.12 ± 0.06	1.26 ± 0.04	−6.7 ± 3.0	***
Lactate_max_ [mmol/l]	4.4 ± 1.3	8.0 ± 1.6	−6.0 ± 2.1	***
RPE_max_ [6–20]	19 ± 1	20 ± 0	− 2.3 ± 2.6	**
Peak torque [Nm]	111 ± 21	173 ± 44	−4.4 ± 3.1	***

Abbreviations: *CI* Confidence interval, *SWD* Smallest worthwhile difference, *BMI* Body mass index, *M*. Musculus, *P*_*max*_ Maximum power, *VO*_*2rest*_ Oxygen uptake at rest, *VO*_*2max*_ Maximum oxygen uptake, *HR*_*max*_ Maximum heart rate, *RER*_*max*_ Maximum respiratory exchange ratio, *RPE*_*max*_ Maximum rating of perceived exertion

Note: Means and 90% CI of both groups and x-fold-SWD are shown. The probabilities that the effects are likely (75 to < 95%), very likely (95 to < 99%), and most likely (≥99%) higher or lower than the SWDs are indicated by the asterisks **, ***, and ****, respectively. If the probabilities that the effects are both higher and lower than the SWDs are of ≥5%, they are unclear as indicated by the letter u

### Study design

A cross-sectional design under laboratory conditions was applied. The following tests were conducted in the mentioned order: (a) anthropometric measurements, (b) incremental test until exhaustion on a cycling ergometer, and (c) maximum isometric strength test of the knee extensors on an isokinetic device. Between the incremental cycling and isometric strength test the participants were given a 30-min break. During all tests, a cardiologist was present and monitored all procedures.

### Anthropometric measurements

Body fat and fat-free mass were determined by using a 4-point bioelectric impedance analysis (Bodystat, QuadScan 4000, Douglas, United Kingdom) in supine position. For later muscle oxygen saturation measures by near-infrared spectroscopy, skinfold thickness of vastus lateralis and biceps brachii muscle was determined using a caliper (Baseline® Medical Skinfold Caliper, Baseline® evaluation instruments, United States). The validity of the 4-point bioelectric impedance analysis is *r* = 0.98–0.99 [29].

### Incremental cycling test

The incremental test was performed on a cycling ergometer (Excalibur sport, Lode, Groningen, Netherlands). Testing consisted of a ramp-like protocol. For reaching a comparable time to exhaustion, the load started and increased per minute by 5 and 10 W for patients with chronic heart failure and healthy controls, respectively. The test was ended, when the required pedalling frequency of 75–80 rpm could no longer be maintained. Finally, patients and healthy controls performed a 5-min cool-down at 15 and 30 W, respectively. To clarify exhaustion, the healthy controls had to reach three out of five criteria: (a) heart rate ≥ 200 bpm – age; (b) blood lactate ≥8 mmol/l; (c) respiratory exchange ratio ≥ 1.1; (d) rating of perceived exertion ≥18; and (e) visual analogue scale ≥60% of total exertion [30–32]. However, as patients with chronic heart failure barely reach blood lactate levels of ≥4 mmol/l and heart rate is affected by beta blockers [30, 33], clarification of exhaustion was based on rating of perceived exertion and the visual analogue scale. During the test, oxygen uptake was measured breath-by-breath and averaged over 60 s using a gas analyzer (Power-Cube Ergo, Ganshorn, Niederlauer, Germany). The gas analyzer was calibrated prior to each testing according to the instructions of the manufacturer. Every minute systolic and diastolic blood pressure were measured manually on the left upper arm. Afterward, mean arterial pressure (MAP) was calculated for every minute as follows:

1 $$ MAP= BPdia+\left(0,33\ast \left( BPsys- BPdia\right)\right) $$,

whereas BPdia and BPsys describe diastolic and systolic blood pressure, respectively. All data collected during the incremental test were interpolated at 60, 70, 80, 90, and 100% of maximum oxygen uptake for later statistical analyses. Immediately after exhaustion, capillary blood was taken from the earlobe to determine the lactate concentration. The samples were analyzed by an electro-enzymatic analyzer (EKF-diagnostics, Biosen C_line Sport, London, United Kingdom). The reliability of the cycling ergometer, gas analyzer, and electro-enzymatic analyzer was reported by a coefficient of variation (CV) = 8.2% [34], intraclass coefficient (ICC) = 0.991–0.995 [35], and CV = 1.3% [36], respectively.

### Bioreactance analysis

Throughout the incremental cycling test, stroke volume (SV) and heart rate (HR) were recorded non-invasively by a bioreactance analysis (Cheetah Nicom, Cheetah Medical, Vancouver, USA). The measurement principle is described in detail elsewhere [37]. Briefly, the technology is based on phase shifts, which occur when an alternating electric current with a frequency of 75 Hz is passed through the thorax. From the phase shifts the stroke volumes are predicted, because both are strongly correlated. According to the instructions of the manufacturer, eight electrodes were applied to the participant’s back prior to testing.

Cardiac output (CO) was estimated afterwards as follows:

2 $$ CO=\frac{HR\ast SV}{1000} $$

Then, based on cardiac output and mean arterial pressure, cardiac power output (CPO) was calculated accordingly:

3 $$ CPO=\frac{CO\ast MAP}{451} $$

All data were measured beat-by-beat and averaged over 60 s.

The validity and reliability of the used bioreactance analysis are regression coefficient (R) = 0.82 [38] and ICC = 0.59–0.98 [39], respectively.

### Near-infrared spectroscopy

Muscle oxygen saturation of the right vastus lateralis muscle was measured using a near-infrared spectroscopy (Moxy Monitor, Fortiori Design LLC, Hutchinson, USA). The measurement principle is also explained in detail elsewhere [40]. Briefly, the technology is based on light waves (630–850 nm), being sequentially send from four light emitting diodes into the tissue beneath. Then, two detectors record the amount of returned scattered light. Surrounded by a light shield, the near-infrared spectroscopy was placed on the prominent part of the muscle belly of vastus lateralis muscle. A second device was applied to the right biceps brachii muscle as a control condition, as conducted before [41]. The biceps brachii muscle was chosen, because of its low activity during cycling [42]. Data were measured at a frequency of 2 Hz and averaged over 60 s. The reliability of the used near-infrared spectroscopy is ICC = 0.773–0.992 [40].

### Isometric strength test

To obtain additional insights into peripheral differences, strength differences between patients with chronic heart failure and healthy controls were examined by a maximum isometric strength test of the knee extensors using an isokinetic device (HUMAC NORM, CSMi solutions, Stoughton, USA). The dominant leg was tested at a hip angle of 90° and a knee angle of 60° flexion and lasted 6 s. The test was performed twice, separated by a 2-min rest period, and the highest peak torque was used for statistical analyses. As body weight was similar in both groups, the data were not relativized for later statistical analyses. The reliability of the used isometric device is ICC = 0.90–0.98 [43].

### Statistical analyses

To investigate differences between both groups, Magnitude-based inferences were computed. This alternative statistical approach is well suited for small sample sizes with high intra- and interindividual variabilities and aims to increase the transfer of findings into practice (i.e., practical before statistical significance). The approach is described in detail elsewhere [44]. Firstly, means and 90% confidence intervals were computed. Next, the usage of the respective confidence intervals in relation to the smallest worthwhile difference (i.e., the pooled standard deviation multiplied by 0.2) were investigated [45]. The probabilities for the differences “truly” being higher, similar, or lower compared to the smallest worthwhile difference were identified and qualitatively described by a probabilistic scale as follows: < 25%, trivial (t); 25 to < 75%, possibly (*); 75 to < 95%, likely (**); 95 to < 99%, very likely (***); and ≥ 99%, most likely (****). In case that the probabilities for being both higher and lower than the smallest worthwhile difference were ≥ 5%, the differences were indicated as unclear (u) [44]. The Magnitude-based inferences were determined using the spreadsheets available at http://www.sportsci.org/. Since hemodynamic responses with the same clinical status may diverge and to emphasize intra- and interindividual variabilities of the participants, individual responses of the underlying factors of maximum oxygen uptake are also shown.

## Results

### Oxygen uptake and rating of perceived exertion

**Fig. 1:**
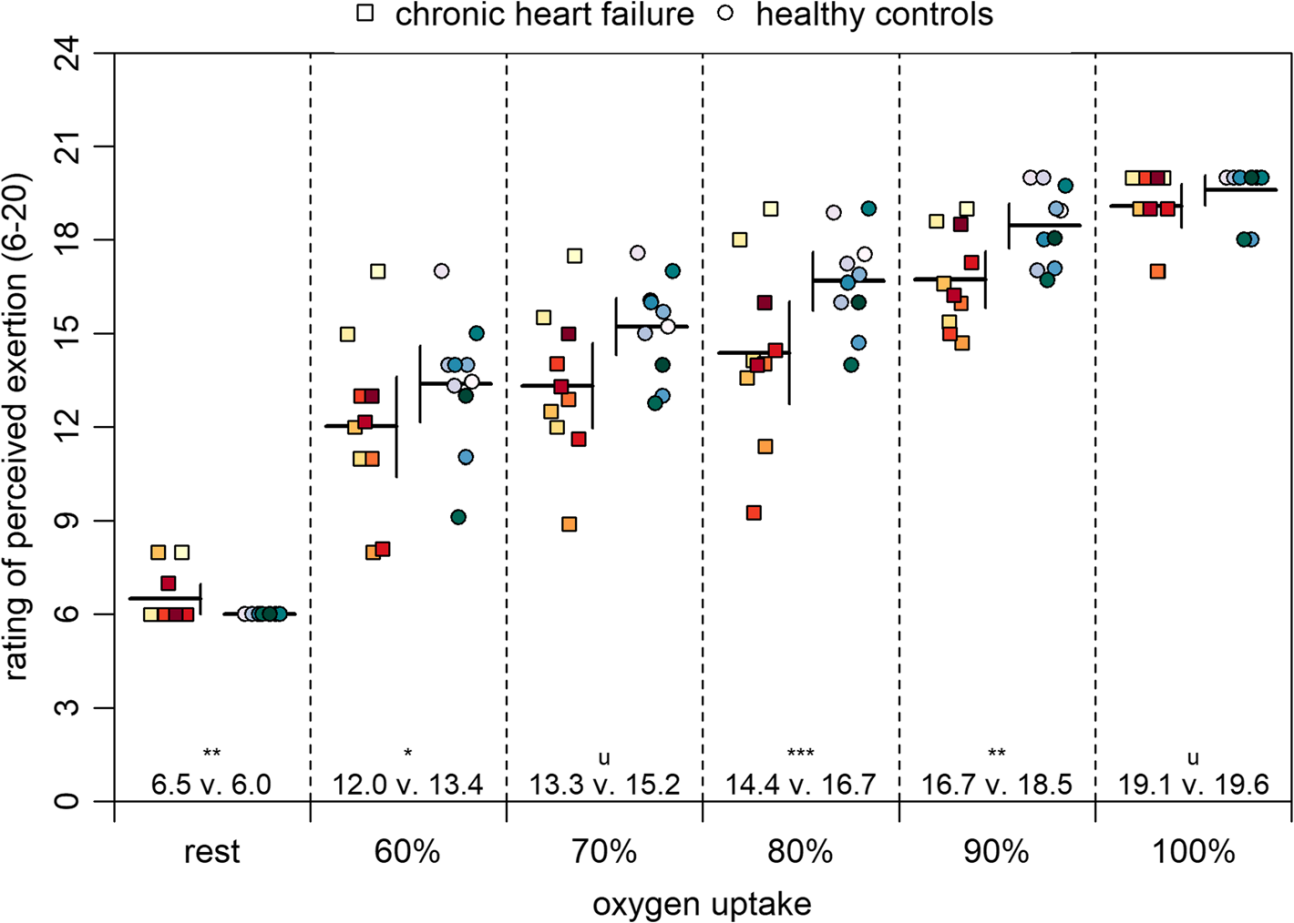
Rating of perceived exertion at rest and at 60 to 100% of maximum oxygen uptake. Note: Means and 90% confidence intervals as well as individual data are shown. The probabilities that the effects are possibly (25 to < 75%), likely (75 to < 95%), and very likely (95 to < 99%) higher or lower than the smallest worthwhile differences are indicated by the asterisks *, **, and ***, respectively. If the probabilities that the effects are both higher and lower than the smallest worthwhile difference are of ≥5%, they are unclear as indicated by the letter u

Table 1 shows that patients with chronic heart failure had a most likely lower maximum oxygen uptake than healthy controls. There were unclear differences at rest. Figure 1 demonstrates that patients had a possibly to very likely lower rating of perceived exertion at 60, 80, and 90% of maximum oxygen uptake. However, at rest, patients had a likely higher rating of perceived exertion and unclear differences were found at 70 and 100% of maximum oxygen uptake.

### Central factors

**Fig. 2:**
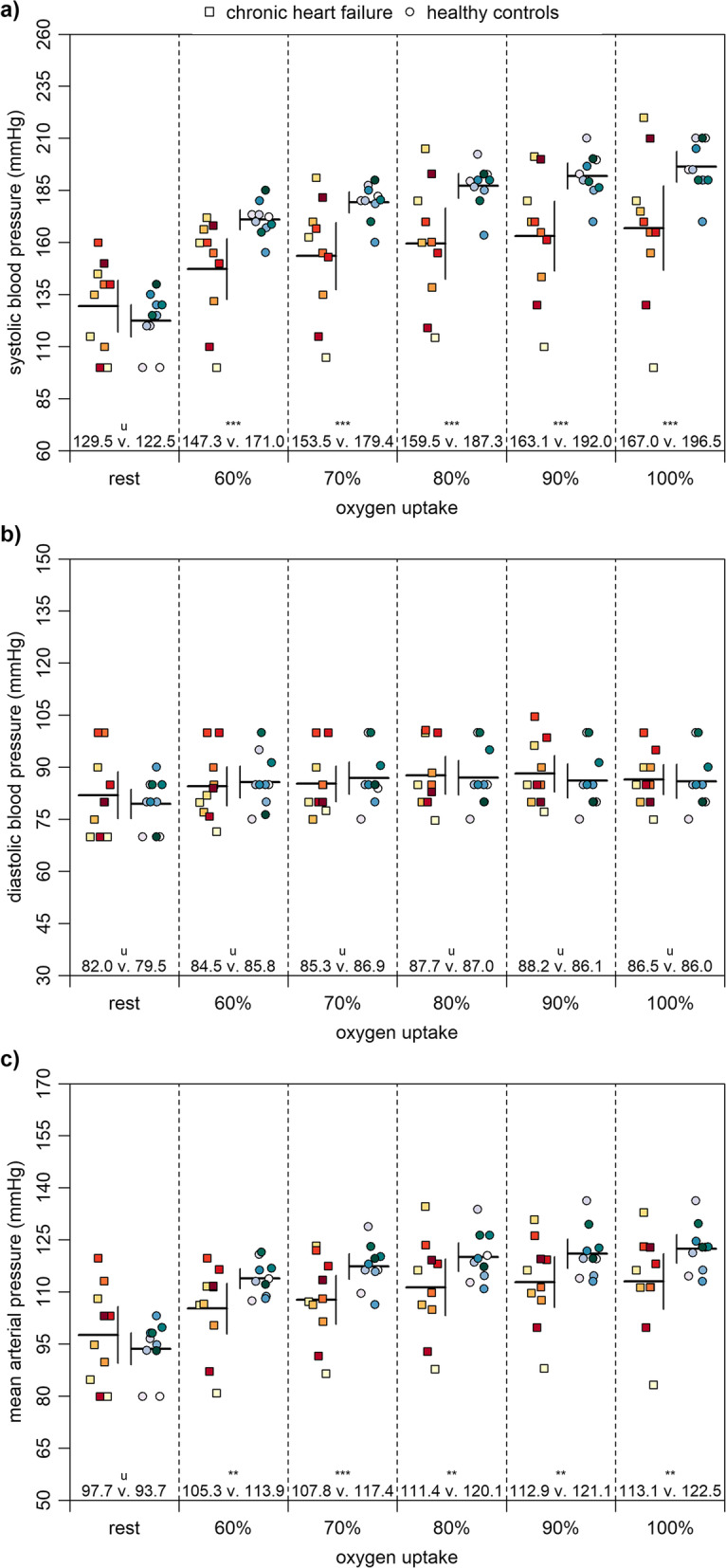
Blood pressure at rest and at 60 to 100% of maximum oxygen uptake. Note: Means and 90% confidence intervals as well as individual data are shown. The probabilities that the effects are likely (75 to < 95%) and very likely (95 to < 99%) higher or lower than the smallest worthwhile differences are indicated by the asterisks ** and ***, respectively. If the probabilities that the effects are both higher and lower than the smallest worthwhile difference are of ≥5%, they are unclear as indicated by the letter u

Figure 2a shows that patients with chronic heart failure had a very likely lower systolic blood pressure at 60–100% of maximum oxygen uptake than healthy controls. There were unclear differences at rest. Figure 2b reveals that there were unclear differences in diastolic blood pressure at 60–100% of maximum oxygen uptake as well as at rest. Figure 2c shows that patients had a likely to very likely lower mean arterial pressure at 60–100% of maximum oxygen uptake. There were unclear differences at rest.

**Fig. 3:**
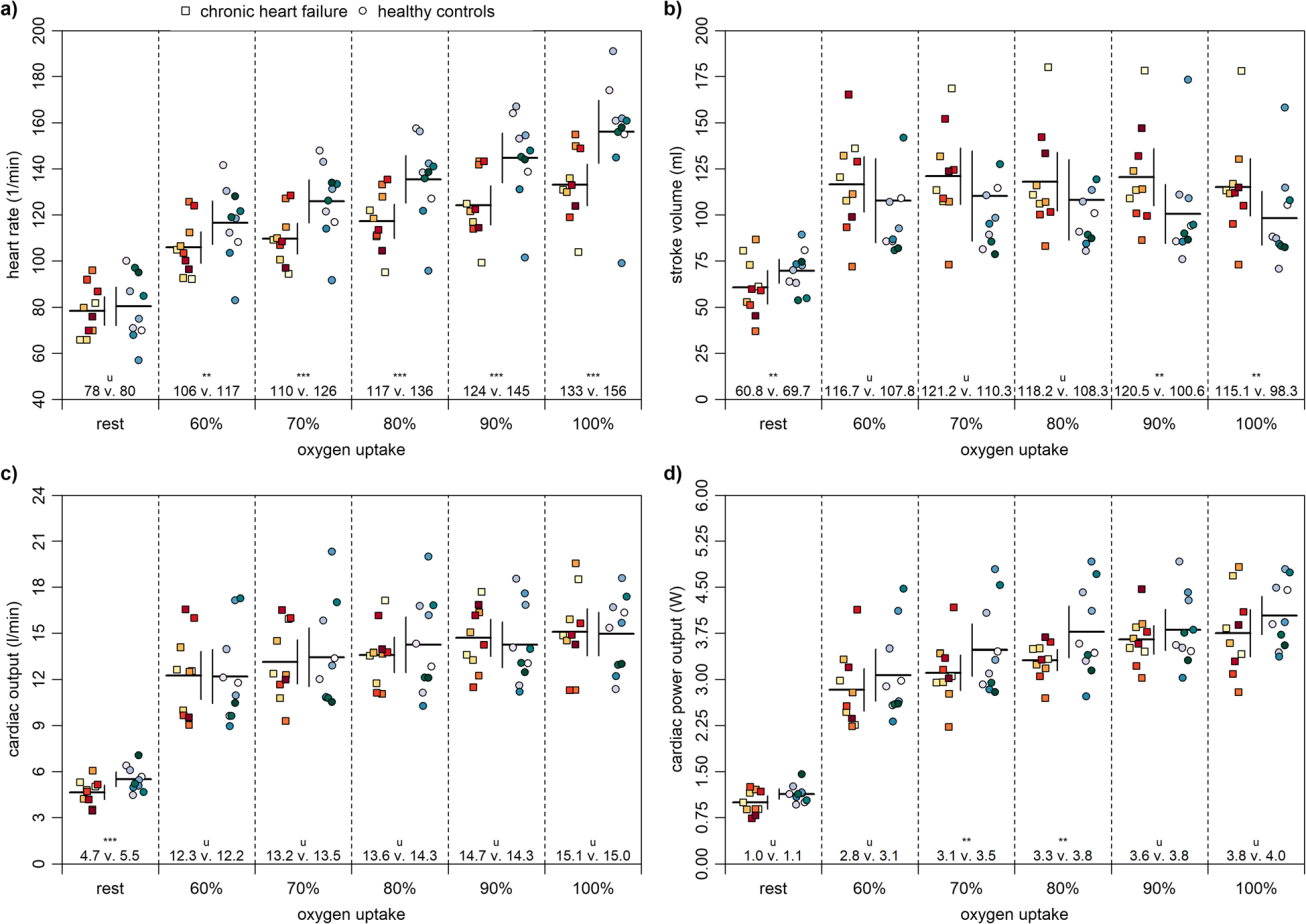
Central factors at rest and at 60 to 100% of maximum oxygen uptake. Note: Means and 90% confidence intervals as well as individual data are shown. The probabilities that the effects are likely (75 to < 95%) and very likely (95 to < 99%) higher or lower than the smallest worthwhile differences are indicated by the asterisks ** and ***, respectively. If the probabilities that the effects are both higher and lower than the smallest worthwhile difference are of ≥5%, they are unclear as indicated by the letter u

Figure 3a shows that patients with chronic heart failure had a likely to very likely lower heart rate at 60–100% of maximum oxygen uptake than healthy controls. There were unclear differences at rest. Figure 3b demonstrates that patients had a likely higher stroke volume at 90 and 100% of maximum oxygen uptake but likely lower at rest. Differences at 60–80% of maximum oxygen uptake were unclear. Figure 3c shows that patients had a very likely lower cardiac output at rest. There were unclear differences at 60–100% of maximum oxygen uptake. Figure 3d reveals that patients had a likely lower cardiac power output at 70 and 80% of maximum oxygen uptake. Differences at 60, 90, and 100% of maximum oxygen uptake as well as at rest were unclear.

### Peripheral factors

**Fig. 4:**
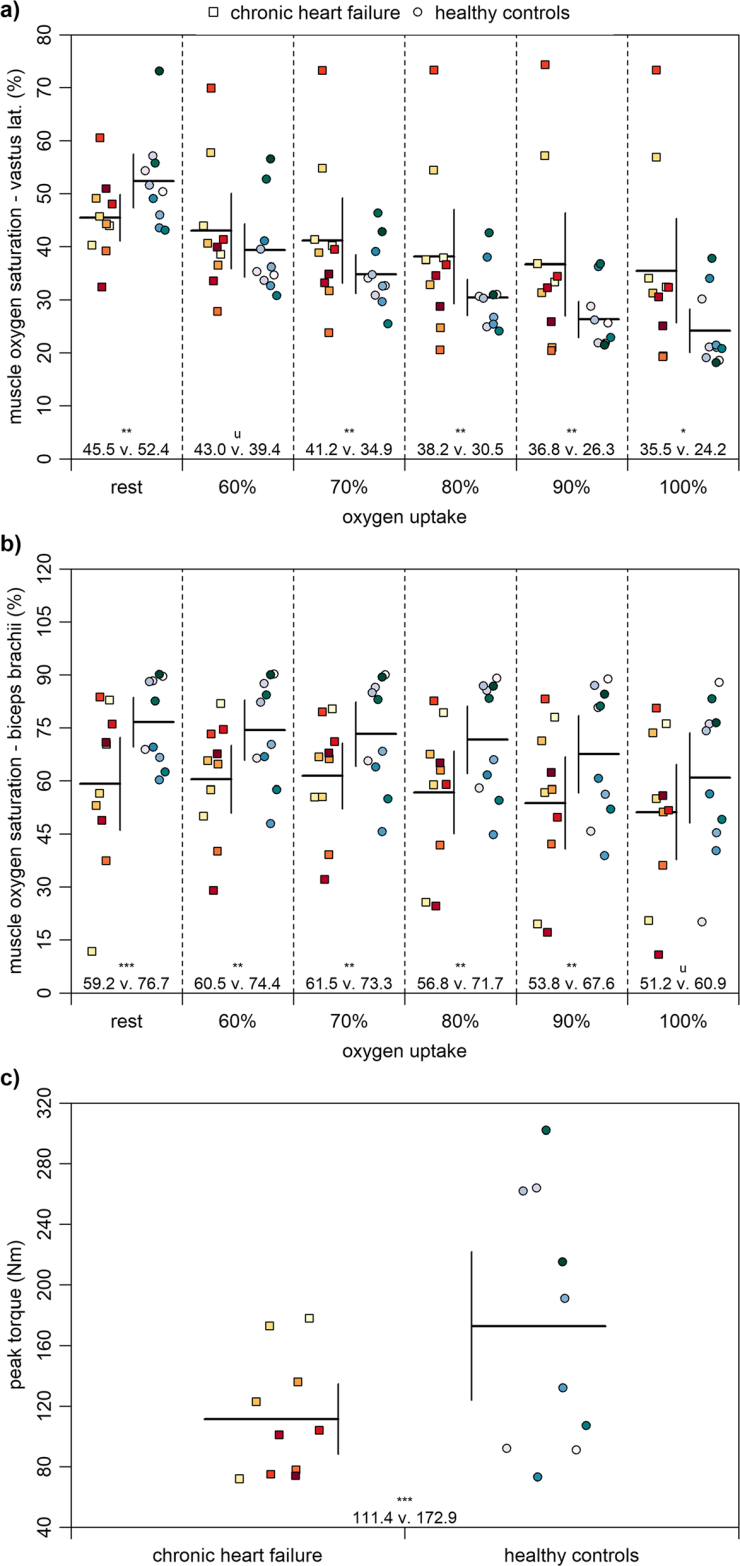
Peripheral factors at rest and at 60 to 100% of maximum oxygen. Note: Means and 90% confidence intervals as well as individual data are shown. The probabilities that the effects are possibly (25 to < 75%), likely (75 to < 95%), and very likely (95 to < 99%) higher or lower than the smallest worthwhile differences are indicated by the asterisks *, **, and ***, respectively. If the probabilities that the effects are both higher and lower than the smallest worthwhile difference are of ≥5%, they are unclear as indicated by the letter u

Figure 4a shows that patients with chronic heart failure had a possibly to likely higher muscle oxygen saturation in the vastus lateralis muscle at 70–100% of maximum oxygen uptake than healthy controls. At rest, patients had a likely lower muscle oxygen saturation in the vastus lateralis muscle. Differences at 60% of maximum oxygen uptake were unclear. Figure 4b reveals that patients had a likely to very likely lower muscly oxygen saturation in the biceps brachii muscle at 60–90% of maximum oxygen uptake as well as at rest. At 100% of maximum oxygen uptake, differences were unclear. Figure 4c shows that patients had a very likely lower maximum torque of the knee extensors of the dominant leg.

## Discussion

For the first time, we investigated non-invasively measured central and peripheral factors of oxygen uptake between patients with chronic heart failure and healthy controls by the same standardized research design. Our main findings were: patients with chronic heart failure had (a) a lower maximum oxygen uptake, (b) a similar cardiac output and cardiac power output at maximum oxygen uptake, and (c) lower values in muscle oxygen saturation of vastus lateralis muscle at rest and higher values at maximum load as well as lower values in isometric peak torque values.

Regarding our first main finding, patients with chronic heart failure had a most likely lower maximum oxygen uptake than healthy controls (Table 1). The maximum oxygen uptake of the patients was 15.6 ± 3.0 ml/kg/min, whereas healthy controls had 28.0 ± 2.1 ml/kg/min (− 44.3%). Previous studies support these findings: 15.4 ± 4.9 vs. 23.1 ± 3.0 ml/kg/min (− 33.3%) [18], 15.2 ± 1.1 vs. 21.1 ± 1.7 ml/kg/min (− 28.0%) [25], and 20.1 ± 6.0 vs. 33.3 ± 7.0 ml/kg/min (− 39.6%) [19]. This outcome reinforces the well-known negative impact of chronic heart failure on maximum oxygen uptake and performance capacity. These differences may be due to several underlying central and peripheral factors. However, solely based on this finding, it is not possible to conclude whether the lower maximum oxygen uptake is primarily impacted by central and/or peripheral factors. Regardless of the potential multifactorial reasons for a lower maximum oxygen uptake, it is still an established parameter for the risk stratification of chronic heart failure [6].

Concerning our second main finding, patients with chronic heart failure had a similar cardiac output and cardiac power output at maximum load compared to healthy controls (Fig. 3). Our results showed a cardiac output for patients and healthy controls of 15.0 ± 1.4 and 15.1 ± 1.3 l/min (− 0.7%), respectively. Values for cardiac power output for patient and healthy controls were 3.8 ± 0.3 and 4.0 ± 0.3 (− 5.0%), respectively. In contrast, previous studies showed lower values of up to 31.0% in cardiac output [10, 11, 20, 21] and up to 45.8% in cardiac power output [10, 11, 22] for patients with chronic heart failure compared to separately measured healthy participants. One possible explanation for these inconsistencies may be the different research designs and the high intra- and interindividual variabilities of the patients with chronic heart failure (Figs. 2 and 3) [15]. Considering that, during load, patients had a lower heart rate but higher stroke volume, our results for cardiac output are plausible. A rational explanation may be that the patients were very well medicated and based on the Kansas City Cardiomyopathy Questionnaire in a well general state. Nevertheless, additional information of the contractile reserve could have been meaningful to clarify our observations [46]. Regarding the risk stratification of chronic heart failure, maximum oxygen uptake is the established gold standard [6]. However, our results show that maximum oxygen uptake reveals little of the actual cardiac performance of patients with chronic heart failure as there are unclear differences in cardiac output and cardiac power output between both groups. These findings show that the cardiac power output may be suited better for estimating the cardiac performance of patients with chronic heart failure [9–11].

Regarding our last major finding, patients with chronic heart failure had lower values in muscle oxygen saturation of the vastus lateralis muscle at rest and higher values at maximum load as well as lower isometric peak torque values compared to healthy controls (Fig. 4). Our results concerning muscle oxygen saturation of the vastus lateralis muscle at rest were 45.5 ± 3.9% and 52.4 ± 4.5% (− 13.2%) for patients and healthy controls, respectively. Another study also found lower values in patients with chronic heart failure at rest, but these were not statistically significant (67.9 ± 4.0% vs. 70.0 ± 5.4%; − 3.0%) [19]. However, in our study, the muscle oxygen saturation of the biceps brachii muscle at rest showed lower values for patients as well (59.2 ± 11.7% vs. 76.7 ± 6.3%; − 22.3%). The muscle oxygen saturation of vastus lateralis muscle at maximum load was higher in patients (35.5 ± 8.8% vs. 24.2 ± 3.7%; + 46.7%), meaning healthy controls may use their oxygen reserves more efficiently compared to patients. The lower muscle oxygen saturation at rest and lower exploitation of oxygen reserves during load of the patients may be caused by the reduced peripheral perfusion, the adaptive mitochondrial dysfunction as well as the shift in muscle-fibre types, whereby slow, oxidative type I fibres are being replaced by fast, glycolytic type IIb fibres [8, 14, 47]. Our results concerning isometric peak torque were 111 ± 21 Nm and 173 ± 44 Nm (− 35.8%) for patients and healthy controls, respectively, and are supported by previous studies, which investigated both groups separately (up to − 35.3%) [23, 24]. The difference between both groups can be explained by the abovementioned peripheral changes, possibly resulting in the lower muscle mass of the patients with chronic heart failure [8, 23]. As mentioned above regarding central factors, peripheral factors also show high intra- and interindividual variabilities (Fig. 3) [15]. Overall, the results show that patients with chronic heart failure have peripheral differences compared to healthy controls, which should be considered in diagnosis and subsequently in therapy. The observed intra- and interindividual variabilities in patients with chronic heart failure could also help to implement therapy on a more individual basis.

Taken together, central and peripheral factors may affect the maximum oxygen uptake in patients with chronic heart failure. Thus, it is promising to measure both types of factors in clinical settings to allow more effective and individually adjusted therapies. The cardiac power output should be gaining increasing importance in diagnosis, follow-ups, and prognosis of heart failure, because of its possible superior prognostic impact compared to maximum oxygen uptake [9–11]. Additionally, peripheral factors should be addressed simultaneously to clarify if a low maximum oxygen uptake is primarily based on central or peripheral factors. This can also be helpful for transplantation decisions in the future [6] for which however more research is needed.

While our study increased the knowledge concerning non-invasively measured central and peripheral factors of oxygen uptake in patients with chronic heart failure, some limitations should be acknowledged. Firstly, we investigated a relatively small sample size, which caused large confidence intervals and unclear differences between the groups. A larger sample size would allow a better generalization. Additionally, a crucial point of our statistical approach is the definition of the smallest worthwhile difference. Compared to sport science, the definition is less approved in sport medical settings [44]. Moreover, the exact etiology of the heart failure of our patients remain unknown and limit more mechanistic pathophysiological discussions [15, 16]. The reason to exclude the etiology was that the potential trigger for the chronic heart failure of our patients was stretching far back into the past. Lastly, it is known that the reliability of the device used for the near-infrared spectroscopy decreases with increasing load [40] and that differences in skinfold thickness between both groups were evident. Thus, differences in maximum load [40] and skinfold thickness [48] between both groups may have had an impact on our near infrared spectroscopy outcomes. Further studies are needed to address these points.

## Conclusions

In conclusion, our study shows that non-invasively measured central and peripheral factors of oxygen uptake differ between patients with chronic heart failure and healthy controls. Therefore, it is promising to measure both types of factors in patients with chronic heart failure to optimize the diagnosis and therapy. Especially, peripheral factors can reveal new insights into the pathophysiology of chronic heart failure and should therefore be more investigated in combination with central factors in future studies.

## Data Availability

The datasets used and/or analyzed during the current study are available from the corresponding author on reasonable request.

## Abbreviations

NYHA: New York heart association
MAP: Mean arterial pressure
BPdia: Diastolic blood pressure
BPsys: Systolic blood pressure
CV: Coefficient of variation
ICC: Intraclass correlation
SV: Stroke volume
HR: Heart rate
CO: Cardiac output
CPO: Cardiac power output
R: Regression coefficient
